# Empowerment in Nursing: A Systematic Review of Its Association With Patient Safety Culture

**DOI:** 10.7759/cureus.107088

**Published:** 2026-04-15

**Authors:** Badria Barnawi, Faridah Mohd Said

**Affiliations:** 1 Nursing, Lincoln University College, Petaling Jaya, MYS

**Keywords:** meta-analysis, nursing empowerment, patient safety culture, psychological empowerment, structural empowerment, systematic review

## Abstract

Empowerment in nursing is increasingly recognized as an important determinant of patient safety culture as the primary outcome, with patient safety activities reported as secondary outcomes where applicable. This systematic review and meta-analysis aimed to synthesize the evidence on the association between nursing empowerment and patient safety culture and to examine whether different types of empowerment were associated with variation in effect estimates. A comprehensive search was conducted in Google Scholar, PubMed, and ScienceDirect, with the final search completed on 6^th^ March 2026. Eligible studies included quantitative primary studies of nurses in clinical settings that reported sufficient data for effect-size estimation. Data were pooled using a random-effects meta-analysis, and subgroup, sensitivity, and reporting-bias analyses were performed. Seven studies met the inclusion criteria, comprising six cross-sectional studies and one randomized controlled trial (RCT), with a total of 1,079 participants. The meta-analysis demonstrated a statistically significant and large pooled association between nursing empowerment and patient safety culture (r = 0.721, 95% CI: 0.424 to 0.878, p < 0.001), although heterogeneity was very high (I² = 98.28%, Q(7) = 460.96, p < 0.001), with a wide prediction interval (r = -0.420 to 0.979). Subgroup analysis showed that psychological empowerment had a significant positive effect (β = 1.2385, p = 0.0022), structural empowerment showed a marginal effect (p = 0.0547), and empowerment programs were not statistically significant (p = 0.2292). Sensitivity analysis indicated that the overall finding was robust, although one study contributed disproportionately to heterogeneity. The funnel plot did not indicate asymmetry upon visual inspection, and Egger's test showed no evidence of publication bias (z = -0.03, p = 0.978). Overall, nursing empowerment was associated with better patient safety culture, but the magnitude and consistency of this association varied across empowerment types and study contexts.

## Introduction and background

Nursing is a high-stakes speciality in which clinical competence and patient safety are paramount. Empowerment at both the organisational and individual levels is believed to enhance nurses' ability to provide safe, effective care. "Structural empowerment" refers to organisational practices that give staff access to resources, information, support, and opportunities [1]. Psychological empowerment describes a nurse's internal sense of meaning, competence, self-determination, and impact in work. When nurses feel empowered, they are more motivated, autonomous, and engaged [2, 3]. Patient safety is a fundamental indicator of healthcare quality. Empowerment is hypothesised to influence patient safety outcomes, particularly through its impact on patient safety culture, by enabling effective teamwork, leadership, and the use of technology [4]. Empowered nurses demonstrate greater willingness to collaborate, share knowledge, and engage in collective learning, behaviours that directly enhance patient safety outcomes [5]. For example, one cross-sectional study found that higher structural empowerment among critical care nurses was associated with stronger patient safety culture, and another reported that psychological empowerment scores correlated positively with nursing interns' clinical competence [6, 7]. This shows that integration of structural and psychological empowerment within healthcare organisations can significantly improve both patient safety and clinical competence. By fostering a culture of empowerment, healthcare systems can promote innovation, reduce medical errors, and enhance the overall quality of care, in line with the Sustainable Development Goals of universal health and well-being [4].

Although patient safety is a multidimensional construct, it is important to distinguish between patient safety culture and patient safety activities, as these terms are often used interchangeably despite representing distinct concepts. "Patient safety culture" refers to the shared values, beliefs, and norms within a healthcare organisation that shape attitudes and behaviours related to safety and is typically assessed using validated instruments such as the Hospital Survey on Patient Safety Culture (HSOPSC). In contrast, patient safety activities refer to observable safety-related behaviours, including adherence to safety protocols, error reporting, and incident prevention practices. Given these conceptual differences, this review focuses primarily on patient safety culture as the main outcome, while studies reporting patient safety activities are interpreted as related but distinct indicators.

Despite this importance, evidence linking empowerment to concrete outcomes in nursing care is fragmented. To address this gap, this review focuses on the association between nursing empowerment and patient safety culture across diverse healthcare settings. Specifically, this study aims to systematically review and synthesise existing evidence on the relationship between nursing empowerment and patient safety culture as the primary outcome across diverse healthcare settings and to determine the strength and consistency of this association. By synthesising international findings, this review aims to inform nursing leadership and policy to improve nursing practice, clinical competence, and patient safety culture, in alignment with global health goals which emphasise safe, effective care.

## Review

Methodology

Study Design

This study used a systematic review and meta-analysis to investigate the connection between nursing empowerment and patient safety culture. The Preferred Reporting Items for Systematic Reviews and Meta-Analyses (PRISMA) criteria were used to conduct the systematic review [8].

Registration

The International Prospective Register of Systematic Reviews (PROSPERO) received a prospective registration of the review protocol. It is registered with the number CRD420261354284.

Eligibility Criteria

The eligibility criteria for the systematic review were predetermined to maintain transparency, consistency, and rigour in selecting studies on the relationship between empowerment and patient safety culture. The inclusion and exclusion criteria are detailed in Table 1.

**Table 1:** Inclusion and exclusion criteria RCT: randomised controlled studies

Criterion	Inclusion criteria	Exclusion criteria
Population	Registered nurses or nursing staff working in clinical healthcare settings, such as hospitals.	Non-nursing populations or mixed professional groups not analysed separately for nurses.
Exposure/Intervention	Studies examining nursing empowerment, including psychological empowerment, structural empowerment, or empowerment-based programmes/interventions.	Studies not addressing empowerment as a key independent variable.
Outcomes	At least one of the following should be included: patient safety culture as the primary outcome and patient safety activities as secondary outcomes.	Studies not reporting outcomes related to patient safety culture or safety practices.
Study design	Quantitative primary studies, including cross-sectional, correlational, RCTs and quasi-experimental designs.	Qualitative studies, reviews, meta-research, editorials, opinions, and conference abstracts.
Statistical data	Studies providing sufficient statistical information for effect-size calculation or conversion to correlation coefficient (r).	Studies with insufficient data for effect-size calculation or interpretation.
Publication type/language	Peer-reviewed, full-text articles published in English.	Non-peer-reviewed publications or studies without full-text availability.

Search Strategy

A comprehensive search was conducted in the electronic databases Google Scholar, PubMed, and ScienceDirect to identify relevant studies examining the relationship between nurse empowerment and patient safety culture. Reference lists of identified studies and institutional repositories were searched. The date of the last search for each source was 6^th^ March 2026. Keywords and Medical Subject Headings (MeSH) terms related to empowerment (nurse empowerment, psychological empowerment, structural empowerment), outcome (patient safety culture, patient safety, safety culture, patient safety activities), and population (nurses, staff nurses, nursing) and setting (hospital, healthcare) were combined using Boolean operators. Limits were set to search for English-language articles only to maintain consistency in data extraction and interpretation. Search strategies for each database are described in Appendix A.

Although additional databases such as Scopus, Web of Science, and Cumulative Index to Nursing and Allied Health Literature (CINAHL) are commonly recommended for systematic reviews, they were not included in this study due to access limitations. However, Google Scholar was used as a broad indexing platform that captures records from multiple databases, including Scopus-indexed and Web of Science-indexed journals. To enhance comprehensiveness, backwards and forward citation tracking of included studies was also performed. Previous methodological evidence suggests that combining PubMed with Google Scholar can achieve high sensitivity in identifying relevant health-related literature. Therefore, while acknowledging this limitation, the search strategy was designed to maximise coverage of relevant studies.

Study Selection and Data Extraction

Two reviewers separately checked the titles and abstracts of every record that was retrieved for eligibility. The inclusion and exclusion criteria were then evaluated for full-text publications of possibly eligible research. Using a standardised data extraction form, two reviewers independently extracted data from the qualifying studies. Study characteristics, sample size, empowerment type, outcome measures, and effect sizes were extracted. Data regarding patient safety culture (primary outcome), with limited evidence on patient safety activities (secondary outcomes) and if available, any correlation or effect size along with study characteristics (author, year, country), sample size, nursing empowerment type (psychological, structural, program-based), intervention details, and funding, if any, were also recorded. Discrepancies were discussed and resolved between the two reviewers.

Quality and Evidence Assessment

The methodological quality of the included studies was assessed using the Appraisal Tool for Cross-Sectional Studies (AXIS) for the cross-sectional studies and the Cochrane risk-of-bias tool for randomized trials (RoB 2) [9]. The AXIS tool addresses quality in domains related to study objectives, methods, results, discussion, and ethical approval. The RoB 2 addresses bias in the domains of the randomisation process, deviations from the intended interventions, missing outcome data, outcome measures, and selection of reported results. Reporting bias was assessed by the visual inspection of asymmetry in funnel plots and results of Egger's test.

Statistical Analysis

Reported statistics were converted to correlation coefficients (r) and subsequently transformed into Fisher’s Z values prior to analysis to stabilise variance and normalise the distribution of effect sizes. The pooled estimates were then back-transformed to correlation coefficients for interpretation.

Where studies reported multiple effect sizes, a single effect size was selected based on conceptual alignment with the primary outcome (patient safety culture) to avoid violation of independence assumptions. In cases where multiple relevant measures were available, effect sizes were averaged to obtain a single estimate per study. The discrepancy between the number of included studies (k = 7) and the number of pooled effect sizes (k = 8) reflects the inclusion of multiple independent estimates from a single study across different empowerment constructs.

A random-effects meta-analysis model was employed using restricted maximum likelihood (REML), assuming true effect sizes vary across studies due to clinical and methodological diversity. The use of a random-effects model is appropriate given the inclusion of studies with different designs (cross-sectional and experimental), populations, and measurement instruments. Combining these designs is methodologically acceptable when estimating overall associations, provided heterogeneity is appropriately accounted for.

Statistical heterogeneity was assessed using Cochran’s Q statistic and quantified using the I² statistic, with values above 75% indicating substantial heterogeneity. To explore potential sources of heterogeneity, subgroup analyses were conducted based on empowerment type (psychological, structural, and program-based).

Sensitivity analysis was performed using a leave-one-out approach to evaluate the influence of individual studies on the overall effect size and heterogeneity estimates.

Publication bias was assessed using visual inspection of funnel plots and Egger’s regression test; however, given the small number of included studies, these results were interpreted with caution.

All analyses were conducted using R software (version 4.5.2, The R Core Team, R Foundation for Statistical Computing, Vienna, Austria) with appropriate meta-analytic packages.

Results

Study Selection

**Figure 1:**
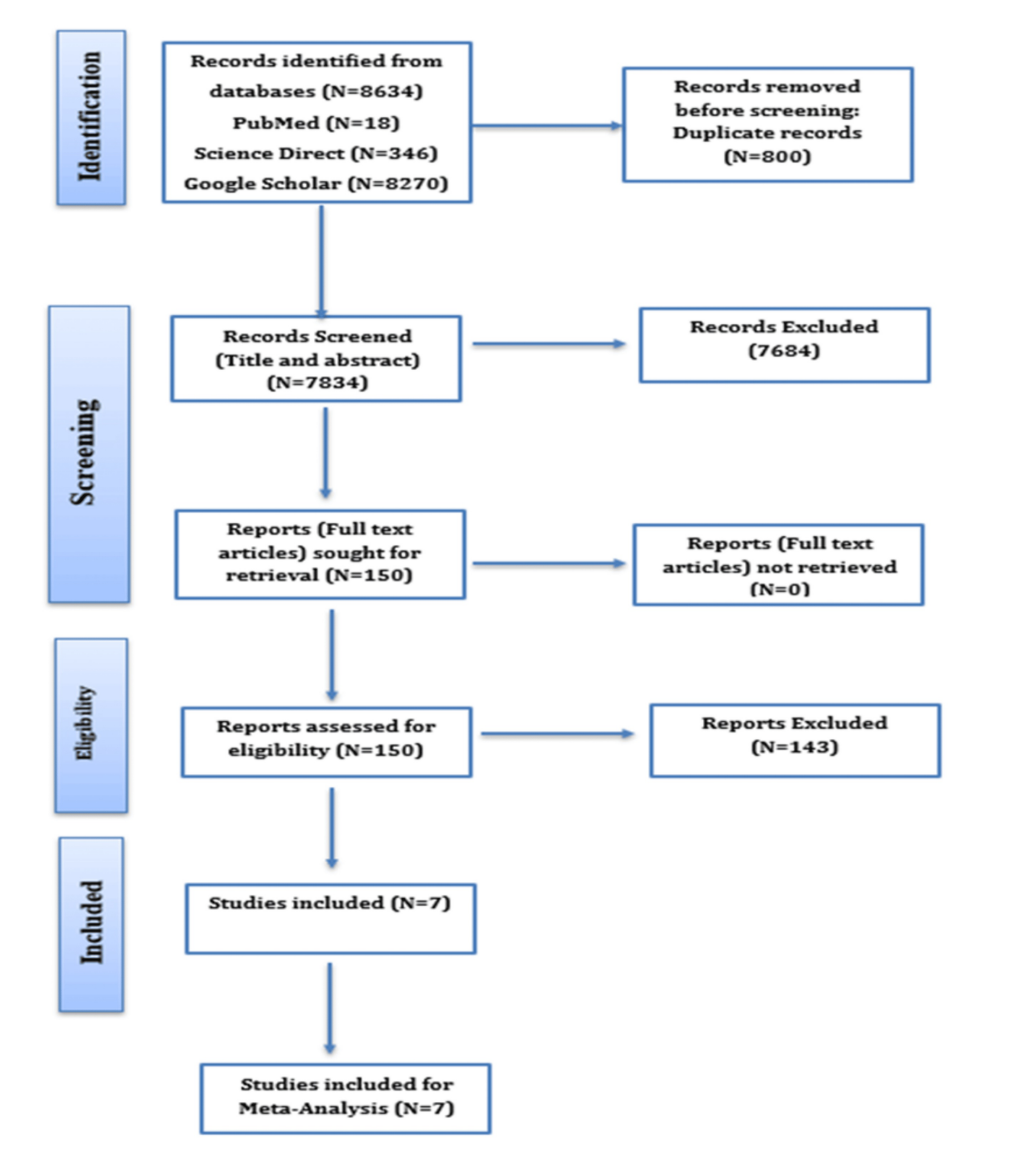
PRISMA Flow Diagram PRISMA: Preferred Reporting Items for Systematic Reviews and Meta-Analyses

Across the three databases, 8,634 records were identified through a search (Google Scholar 8,270, PubMed 18, ScienceDirect 346). We removed 800 duplicate records, and 7,470 records were screened based on title and abstract. Eligibility criteria were applied to exclude 7,320 records, leaving 150 full-text articles to be assessed for eligibility. A total of 143 papers were excluded after full-text assessment due to irrelevant outcome, ineligible population, no empowerment outcome, or lack of empowerment data. Finally, seven studies were included in the systematic review, and all the studies were included in the meta-analysis. Some studies that initially appeared to be eligible were later excluded because they examined empowerment of nurses in a non-clinical setting, reported only qualitative findings, or lacked sufficient statistical data. A flow diagram of study selection is provided in Figure 1, illustrating the process of identification, screening, eligibility, and inclusion in the meta-analysis.

Study Characteristics

Seven studies met the inclusion criteria and were included in the analysis (Table 2). Two studies were from Indonesia, one from Iran, one from South Korea, one from Egypt, one from Turkey and one from the United States. Six of the studies were cross-sectional, correlational, and one was a randomised controlled trial (RCT) [6,10-15].

**Table 2:** Characteristics of the included studies RCT: randomised controlled trial; HSOPSC: Hospital Survey on Patient Safety Culture; PSC: Patient Safety Culture; CONSORT: Consolidated Standards of Reporting Trials; RNs: registered nurses; CWEQ-II: Conditions of Work Effectiveness Questionnaire-II; K-JCAT: Korean Just Culture Assessment Tool; AVE: average variance extracted; PLS-SEM: partial least squares structural equation modelling;

Author and Publication Year	Country	Purpose / Aim of the Study	Study Design	Sample Size	Data Collection Instruments	Intervention	Key Findings	Strengths	Limitations
Amiri et al. [15] (2018)	Iran	To determine the effect of empowering nurses and supervisors through an educational program on patient safety culture in adult ICUs	RCT with pre-test and post-test control groups	61 participants	• HSOPSC (Persian version; 42 items; α=0.84)• Single item on patient safety grading	Educational empowerment program: two-day workshop, posters, weekly pamphlets	• Intervention group: total PSC improved from 2.91±0.40 → 3.46±0.26 (p<0.001)• Control group: no significant change	• RCT design with randomisation • Use of validated, reliable instrument (HSOPSC; α=0.84) • Groups homogeneous at baseline • Effect sizes calculated (Cohen's d) • CONSORT diagram provided • Intervention included both nurses and supervisors	• Single-center study limits generalizability • Supervisors did not provide direct patient care • Three-month follow-up may not capture sustainability • Self-reported instrument used
Armellino et al. [13] (2010)	United States (New York)	To examine the relationship between structural empowerment and PSC in adult critical care RNs	Descriptive correlational	102 RNs	• CWEQ-II (α=0.60–0.90)• HSOPSC (α=0.63–0.84)	No intervention	• Moderate structural empowerment (20.55)• Positive correlations with PSC dimensions	• First study in adult critical care units using HSOPSC • Use of validated, reliable instruments • Clear theoretical framework • Adequate sample size determined by a priori power analysis	• Cross-sectional design limits causal inference • Single tertiary hospital limits generalizability • Low response rate (40%) may affect validity • Relatively low Cronbach's α for formal power (0.60) and staffing (0.63)
Çınar and Kutlu [14] (2021)	Turkey (Istanbul, two hospitals)	To determine the effect of structural & psychological empowerment on patient & employee safety culture	Relational cross-sectional	153 surgical nurses	• CWEQ-II (α=0.69–0.90)• Spreitzer Psychological Empowerment Scale• PSC & Employee Safety Scales	No intervention	• Structural & psychological empowerment positively correlated with PSC & employee safety culture• Structural β=0.498, Psychological β=0.430	• Examination of both structural and psychological empowerment together • Focus on both patient and employee safety culture • Adequate sample size (n=153) • High correlation coefficients	• Cross-sectional design limits causal inference • Single city, two public hospitals limit generalizability • Surgical nurses only • Relatively low Cronbach's α for structural empowerment sub-dimensions (0.69)
Kim and Yu [10] (2021)	South Korea	To examine hospital nurses' perceptions of just culture, empowerment, and patient safety activities	Cross-sectional survey	189 nurses	• K-JCAT (24 items; α=0.72)• Spreitzer Psychological Empowerment Scale (16 items; α=0.93)• Patient Safety Activities Tool (40 items; α=0.92)	No intervention	• Positive correlations: just culture & empowerment (r=0.427); just culture & patient safety activities (r=0.369); empowerment & patient safety activities (r=0.380)• Empowerment & just culture explained 19.5% variance in patient safety activities	• Adequate sample size determined by G*Power • Use of validated instruments with good reliability • Comprehensive analysis including multiple linear regression • Clear conceptual framework provided	• Cross-sectional design limits causal inference • Single geographic region limits generalizability • Convenience sampling may introduce selection bias
Rusdi et al. [11] (2024)	Indonesia (Samarinda Regional Hospital)	To examine the effect of empowerment on patient safety culture among hospital nurses	Cross-sectional, descriptive	119 nurses	• CWEQ-II (α=0.973)• HSOPSC (42 items, 12 dimensions)	No intervention	• Empowerment positively correlated with PSC (β=0.677, p<0.001)• R²=0.458	• Use of validated instruments with excellent reliability (Cronbach's α=0.973; AVE=0.859) • Sample size calculated using Slovin formula • PLS-SEM analysis used • Clear geographical mapping of study location provided (4 map perspectives) • Ethical approval obtained from Samarinda Hospital Ethics Committee	• Cross-sectional design limits causal inference • Single hospital setting limits generalizability • Convenience sampling may introduce selection bias • Only inpatient ward nurses included; excludes ICU, emergency, and outpatient nurses • Self-reported data may be subject to social desirability bias
Umar et al. [6] (2022)	Indonesia (Banten)	To examine the relationship between empowerment and PSC among nurses	Cross-sectional	150 nurses	• Spreitzer Psychological Empowerment Scale (α=0.86)• HSOPSC (42 items, α=0.85)	No intervention	• Moderate empowerment (3.55/5)• Moderate PSC (3.72/5)• Empowerment predicted PSC (β=0.98, p=0.001)	• Validated instruments• Ethical approval obtained• Regression analysis	• Cross-sectional design limits causal inference • Single public hospital setting in Banten limits generalizability • Convenience sampling may introduce selection bias • Self-reported data may be subject to social desirability bias
Youssef et al. [12] (2025)	Egypt (Fowa Central Hospital)	To determine relationships between structural empowerment and PSC	Descriptive correlational	225 staff nurses	• CWEQ-II (α=0.80)• PSC Questionnaire (α=0.79)	No intervention	• 78.2% moderate structural empowerment• PSC moderate (mean 133.43±9.80)• Empowerment & PSC positively correlated (r=0.30)	• Adequate sample• Validated instruments• Pilot study	• Cross-sectional• Single hospital• Self-reported

The sample size ranged from 61 to 225, and the participants were hospital-based registered nurses, including those from speciality units like intensive care and surgical units. The studies examined various aspects of empowerment in nursing as their focus. Psychological empowerment was measured in Kim and Yu [10] and Umar et al. [6]. Structural empowerment was examined in several studies (Rusdi et al., [11]; Youssef et al., [12]; Armellino et al., [13]). One study focused on psychological and structural empowerment (Çınar & Kutlu, [14]), and another on an educational empowerment program (Amiri et al., [15]). Other instruments used for data collection in these studies included the Spreitzer Psychological Empowerment Scale, Conditions of Work Effectiveness Questionnaire (CWEQ-II), and the Hospital Survey on Patient Safety Culture (HSOPSC). All but one of these studies were observational and non-interventional; the exception was an educational empowerment program using workshops and other educational materials.

In summary, the selected studies reflect the relationship between various types of nursing empowerment and patient safety culture, patient safety actions, and clinical competency in a wide range of healthcare settings.

Quality and Evidence Assessment

A critical appraisal of included studies is presented in Table 3. The cross-sectional studies (Kim & Yu, [10]; Rusdi et al., [11]; Youssef et al., [12]; Çınar & Kutlu, [14]; Armellino et al., [13]; Umar et al., [6]) had clearly stated aims, appropriate designs, and valid and reliable measurements, with an overall high quality. The primary methodological issue noted was selection bias, which was not discussed in most studies except for Rusdi et al. [11], and minor sampling and reporting bias issues were observed in Umar et al. [6]. Low risk of bias was found in most domains for the randomised controlled trial conducted by Amiri et al. [15]. Because it was not a blinded study, some bias is likely, and probably some bias is present due to unclear allocation concealment (Table 4).

**Table 3:** Critical appraisal of the included studies (cross-sectional studies) AXIS: Appraisal Tool for Cross-Sectional Studies

Appraisal Criteria (AXIS tool)	Kim & Yu [10] (2021)	Rusdi et al. [11] (2024)	Youssef et al. [12] (2025)	Çınar & Kutlu [14] (2021)	Armellino et al. [13] (2010)	Umar et al. [6] (2022)
1. Is the study aim clearly stated?	Yes	Yes	Yes	Yes	Yes	Yes
2. Is a cross-sectional design appropriate for the aim?	Yes	Yes	Yes	Yes	Yes	Yes
3. Was sample size justification reported?	Yes	Yes	Yes	No	Yes	Yes
4. Is the target population clearly defined?	Yes	Yes	Yes	Yes	Yes	Yes
5. Is the sampling frame representative of the target population?	Yes	Yes	Yes	Yes	Yes	No
6. Was the sample selection method clearly described and appropriate?	Yes	Yes	Yes	Yes	Yes	No
7. Is the risk of selection bias minimised?	No	Yes	No	No	No	No
8. Were the measurements appropriate and valid?	Yes	Yes	Yes	Yes	Yes	Yes
9. Were the measurements reliable?	Yes	Yes	Yes	Yes	Yes	Yes
10. Are the statistical methods clearly described?	Yes	Yes	Yes	Yes	Yes	Yes
11. Are the methods sufficiently described to enable repetition?	Yes	Yes	Yes	Yes	Yes	Yes
12. Are the basic data adequately described?	Yes	Yes	Yes	Yes	Yes	No
13. Is the response rate reported and acceptable?	Yes	Yes	Yes	Yes	Yes	Yes
14. Is information on non-responders provided?	No	No	Yes	Yes	Yes	Yes
15. Are the results internally consistent?	Yes	Yes	Yes	Yes	Yes	No
16. Are all the results described that were stated in the methods?	Yes	Yes	Yes	Yes	Yes	Yes
17. Are the conclusions justified by the results?	Yes	Yes	Yes	Yes	Yes	Yes
18. Are the study limitations discussed?	Yes	Yes	Yes	Yes	Yes	No
19. Are conflicts of interest declared?	Yes	Yes	Yes	Yes	Yes	Yes
20. Is ethical approval reported?	Yes	Yes	Yes	Yes	Yes	Yes

**Table 4:** Critical appraisal of the included studies that are randomised controlled trials RoB 2: Cochrane risk-of-bias tool for randomized trials

Bias Domain (Amiri et al., 2018) [15] (RoB 2)	Low	High	Unclear
Random sequence generation	✔		
Allocation concealment			✔
Blinding (participants/personnel)		✔	
Blinding (outcome assessment)	✔		
Incomplete outcome data	✔		
Selective reporting	✔		
Other bias	✔		

Meta-Analysis

**Figure 2:**
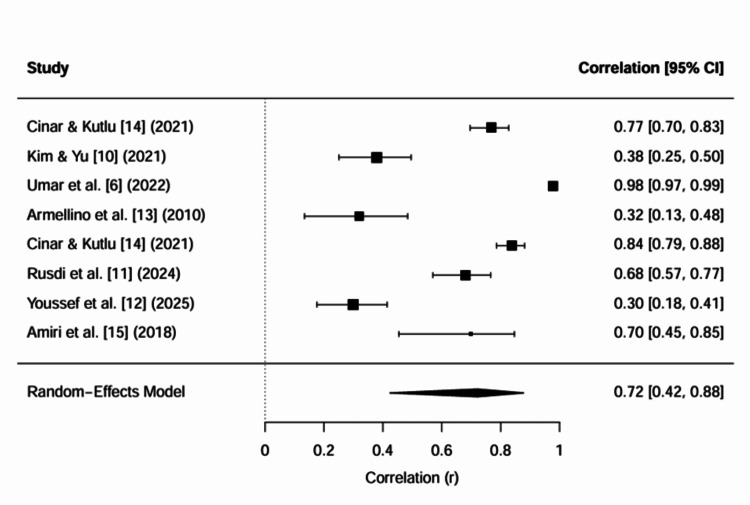
Meta-analysis diagram (nurse empowerment effect of patient safety culture)

A random-effects meta-analysis demonstrated a statistically significant and large pooled effect size (Z = 0.9099, SE = 0.2334, p < 0.001), corresponding to a back-transformed correlation of r = 0.721 (95% CI: 0.424 to 0.878). This indicates a strong positive association across the included studies. However, heterogeneity was extremely high (I² = 98.28%, Q(7) = 460.96, p < 0.001), and the wide prediction interval (r = -0.420 to 0.979) suggests considerable variability in true effects across different contexts (Figure 2).

The observed high heterogeneity likely reflects substantial variability across the included studies in terms of study design, measurement instruments, and contextual factors. Specifically, the included studies comprised predominantly cross-sectional designs alongside one randomised controlled trial, utilised different tools to assess patient safety culture and related constructs (e.g., HSOPSC versus safety activity scales), and examined diverse forms of empowerment (psychological, structural, and program-based). Additionally, variations in healthcare systems and organisational contexts across countries may have contributed to differences in effect sizes.

To explore this variability, a mixed-effects subgroup analysis was conducted (Table 5). The test of moderators was statistically significant (QM(3) = 14.54, p = 0.0023), indicating that empowerment type explains some of the between-study differences. Specifically, psychological empowerment showed a significant and strong positive effect (β = 1.2385, p = 0.0022), while structural empowerment demonstrated a marginal effect (p = 0.0547). In contrast, empowerment programs did not yield a statistically significant effect (p = 0.2292) (Table 6). Despite this, residual heterogeneity remained substantially high (I² = 98.63%), suggesting that, although empowerment type explains part of the variability, a substantial proportion of heterogeneity remains attributable to unmeasured factors such as organisational culture, leadership practices, staffing levels, and differences in safety measurement frameworks across settings (Table 5).

**Table 5:** Summary of mixed-effects meta-analysis model and heterogeneity statistics

Parameter	Number of Studies(k)	Model	Tau² (Residual)	Tau (Residual)	I² (Residual)	H²	QE (df = 5)	QE p-value	QM (df = 3)	QM p-value
Value	8	Mixed-effects (REML)	0.4833	0.6952	98.63%	73.02	388.943	< 0.001	14.535	0.0023

**Table 6:** Moderator analysis of empowerment type in the mixed-effects meta-analysis

Empowerment Type	Estimate (β)	SE	Z-value	p-value	95% CI
Empowerment Program	0.8673	0.7213	1.2024	0.2292	-0.5465 to 2.2811
Psychological	1.2385	0.4040	3.0658	0.0022	0.4467 to 2.0302
Structural	0.6729	0.3503	1.9211	0.0547	-0.0136 to 1.3594

Sensitivity analysis using the leave-one-out approach (Table 7) confirmed the robustness of the overall findings, as the pooled effect size remained statistically significant across all iterations. However, the removal of Umar et al. [6] resulted in a notable reduction in heterogeneity (I² decreased to 94.41%; τ² = 0.1285), indicating that this study contributed disproportionately to between-study variability.

**Table 7:** Results of sensitivity analysis

Study Removed	Estimate	SE	Z-value	p-value	95% CI	Tau²	I² (%)	Q
Çınar & Kutlu (2021) [14]	0.8940	0.2688	3.3253	0.0009	0.3671 to 1.4209	0.4948	98.48	457.72
Kim & Yu (2021) [10]	0.9837	0.2559	3.8435	0.0001	0.4821 to 1.4853	0.4473	98.26	408.55
Umar et al. (2022) [6]	0.7068	0.1409	5.0155	<0.001	0.4306 to 0.9830	0.1285	94.41	121.68
Armellino et al. (2010) [13]	0.9926	0.2520	3.9396	0.0001	0.4988 to 1.4864	0.4338	98.37	427.78
Çınar & Kutlu (2021)	0.8650	0.2645	3.2703	0.0011	0.3466 to 1.3835	0.4787	98.43	441.18
Rusdi et al. (2024) [11]	0.9215	0.2690	3.4259	0.0006	0.3943 to 1.4487	0.4956	98.54	460.57
Youssef et al. (2025) [12]	0.9971	0.2504	3.9811	0.0001	0.5062 to 1.4879	0.4277	98.12	369.17
Amiri et al. (2018) [15]	0.9156	0.2672	3.4271	0.0006	0.3920 to 1.4393	0.4927	98.68	460.95

Assessment of Risk of Bias

**Figure 3:**
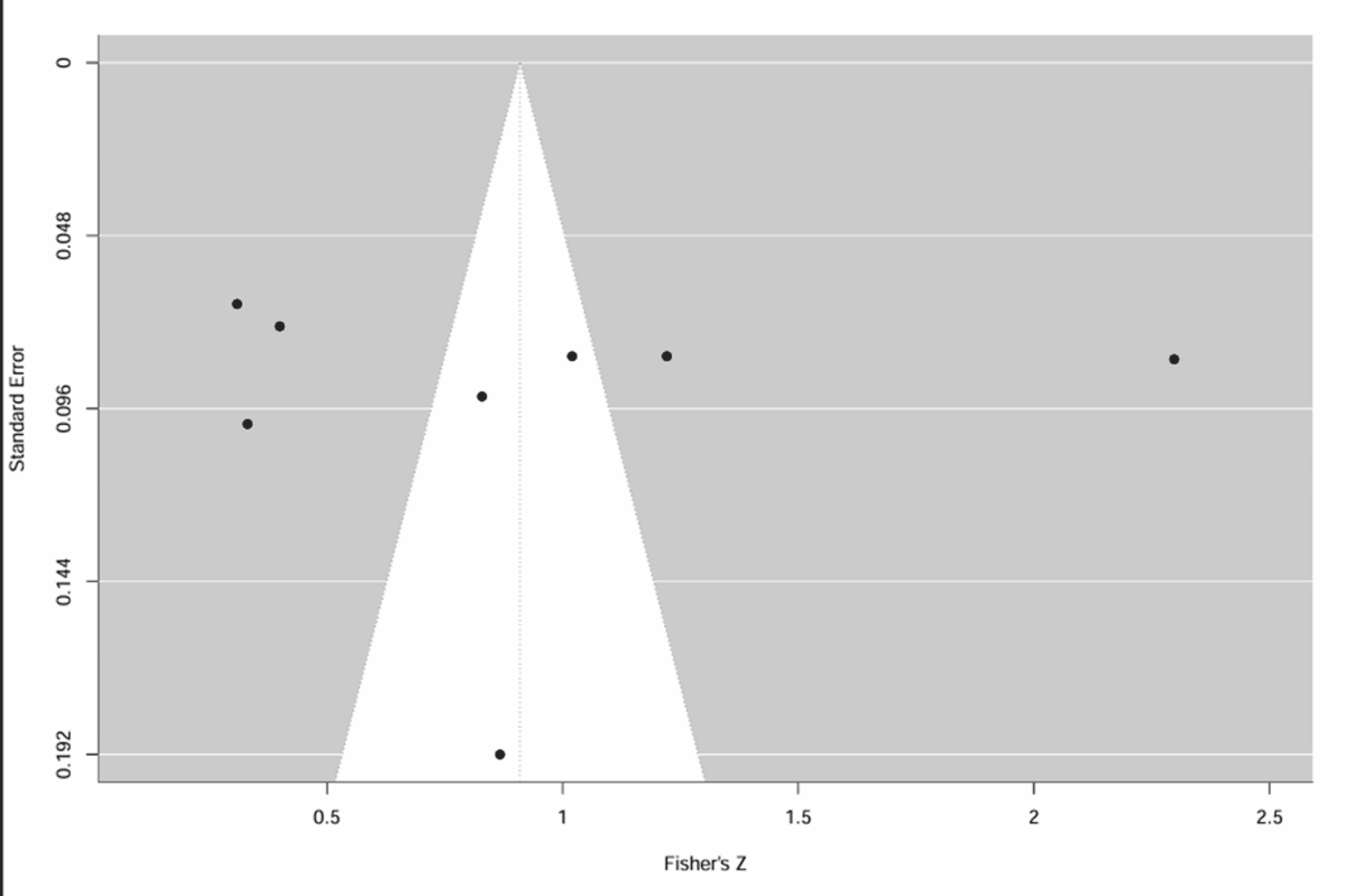
Funnel plot assessing publication bias among the included studies. The plot displays the distribution of individual study effect sizes against their corresponding standard errors. Visual inspection indicates a broadly symmetrical distribution of studies around the pooled effect estimate, suggesting no substantial evidence of publication bias. Although some dispersion of studies is observed, this is likely attributable to heterogeneity and the relatively small number of included studies. This interpretation is further supported by the results of Egger’s regression test (z = −0.03, p = 0.978), which did not demonstrate statistically significant asymmetry.

Reporting bias was assessed by the visual inspection of asymmetry in funnel plots and results of Egger's test. The funnel plot (Figure 3) did not indicate asymmetry upon visual inspection, although the studies appeared to be scattered. Egger's test results indicated that there was no significant publication bias (z = −0.03, p = 0.978). Due to the small number of studies included and the variability of effect sizes, results were interpreted cautiously.

Discussion

This systematic review and meta-analysis examined the association between nursing empowerment and patient safety culture across diverse healthcare settings. Seven studies were included, encompassing data from registered nurses in clinical settings across Indonesia, Iran, South Korea, Egypt, Turkey, and the United States. The reviewed studies investigated psychological empowerment, structural empowerment, and educational empowerment programs, and their relationships with patient safety culture and patient safety activities.

Overall, the meta-analysis demonstrated a significant and strong positive correlation between nursing empowerment and perceptions of patient safety culture, indicating that nurses who feel more empowered tend to report stronger safety cultures. This moderate, statistically robust effect, despite considerable heterogeneity, suggests that empowering work environments consistently enhance safety attitudes. Moreover, the subgroup analysis revealed that psychological empowerment demonstrated a significant and strong positive effect, while structural empowerment also showed a significant but marginal positive correlation with safety culture. In contrast, standalone empowerment programs did not yield a statistically significant effect. In sum, this review highlights the overall contribution of empowerment to patient safety culture, with nurses' intrinsic empowerment (autonomy, meaning, confidence) proving more influential than short-term training interventions. These results advance previous work by quantitatively confirming that empowered nurses support safer care environments, underscoring the critical role of empowerment in fostering a culture of patient safety.

The review's conclusions are in line with earlier empirical studies that show how nurse empowerment improves patient safety outcomes. In a study including 1,026 Chinese nurses, Xu et al. [16] investigated the moderating influence of structural empowerment on the connection between burnout and patient safety culture. They discovered that structural empowerment significantly moderated patient safety culture. Similarly, Kim and Yu [10] surveyed 189 hospital nurses and found that empowerment correlated positively with nurses' patient safety activities. Çınar and Kutlu [14] also reported that surgical nurses with higher structural and psychological empowerment perceived significantly better patient safety culture. Mechanistically, empowerment appears to encourage nurses' voice, engagement and autonomy, which in turn promotes error reporting, teamwork, and vigilance (core aspects of safety culture), as empowered nurses are more likely to engage in safety activities and speak up about threats [10]. Taken together, these empirical findings support the meta-analysis result that higher empowerment is associated with higher patient safety, providing quantitative evidence that nursing empowerment is a significant predictor of a positive safety culture, even if the exact effect sizes vary.

Subgroup analyses shed light on how different types of empowerment relate to safety culture, as the test of moderators indicated significant differences by empowerment type. The particularly strong effect of psychological empowerment observed in this review is supported by recent empirical evidence. A cross-sectional study examining the relationship between nursing practice environment and psychological empowerment among over 2,000 nurses found significant positive correlations between empowerment and professional practice environment characteristics, demonstrating that nurses working in environments with high Magnet hospital characteristics report higher levels of psychological empowerment [17]. This finding is further corroborated by a study among Saudi Arabian nurses, which found that nurses with the highest psychological empowerment demonstrated the greatest flexibility in clinical decision-making [18]. Additionally, a study that looked at the relationship between psychological empowerment and perceived management commitment to safety among Jordanian emergency nurses discovered that psychological empowerment strengthens nurses' capacity to effectively provide patient safety by encouraging them to follow and participate in safety measures [19]. These findings align with the meta-analysis's strong positive β for psychological empowerment.

The marginal effect of structural empowerment on patient safety culture identified in this review also finds support in the broader literature. Recent empirical studies suggest that structural empowerment may support patient safety culture most clearly when it is embedded in stronger practice environments. In Jordan, facets of structural empowerment (staffing and resource adequacy, nurse-manager support, and participation in hospital affairs) were associated with higher perceived patient safety, with staffing/resource adequacy and nurse-manager ability showing the strongest correlations [20]. Similarly, Polish nurses reported better safety when human and material resources, managerial support, nurse-physician cooperation, and participation in management were stronger [21]. More recently, structural empowerment was shown to moderate the burnout-patient safety culture relationship, and Magnet-recognised hospitals were associated with more favourable patient safety and work-environment ratings, indicating that structural empowerment may yield smaller or more conditional effects when organisational support is uneven [16,22]. Overall, these recent studies echo the meta-analysis's marginal p-value, suggesting structural empowerment helps safety culture but may yield smaller or less consistent effects.

The non-significant effect of empowerment programs observed in this review suggests important nuances in how different empowerment dimensions translate into safety culture outcomes. A scoping review of interventions to support nurses' psychological empowerment found that, while various educational programs showed promise, their effects were not uniformly significant across all empowerment dimensions [23]. Çınar and Kutlu [14] found that both structural and psychological empowerment independently predicted better safety culture (beyond burnout) among perioperative nurses, whereas brief classroom-style interventions are often insufficient to change long-term culture on their own. One explanation is that ingrained empowerment (through participative decision-making and work conditions) has a broader impact on the work environment, whereas isolated training may not readily translate to sustained improvements in safety culture.

Contrary to the meta-analysis's pooled non-significant effect, Suryani et al. [24] found that a comprehensive three-month empowerment programme did significantly raise nurses' safety culture scores, suggesting that intensive, multi-modal empowerment programs can succeed. Similarly, Yılmaz and Duygulu [25] showed that an empowerment-based training increased empowerment subscales and safety culture metrics. Methodological factors may also contribute to this disparity: programs often had small samples and varying implementation fidelity, which may explain why this review's pooled analysis of programme studies lacked significance. Nevertheless, the present meta-analysis suggests that routine measures of empowerment (as a trait or structural resource) are more consistently linked to safety culture than standalone interventions. The consistent positive effects of psychological and structural empowerment in the subgroup analysis underscore that organisations should focus on creating empowering environments to enhance safety, in line with prior research.

Importantly, sensitivity checks reinforced the robustness of this review's findings. A leave-one-out analysis showed that no single study disproportionately influenced the pooled correlation. Likewise, evaluation of publication bias (funnel plot and Egger's test) found no evidence of systematic bias. Thus, the positive empowerment-safety correlation appears stable across study contexts. The magnitude of heterogeneity does suggest variability in context and measures; this is likely due to differences in healthcare settings, empowerment instruments, and safety culture scales across studies, as seen in previous meta-analytic research in this domain [26]. The extremely high heterogeneity observed in this meta-analysis warrants careful interpretation of the pooled effect size. This level of variability suggests that the strength of the association between empowerment and patient safety culture is highly context-dependent rather than uniform across settings. Differences in study design (cross-sectional versus experimental), measurement tools (validated safety culture instruments versus behavioural safety activity scales), and conceptualisation of empowerment (psychological versus structural) likely contributed substantially to the observed dispersion in effect sizes. Furthermore, contextual differences across healthcare systems, including organisational structure, leadership models, and resource availability, may influence how empowerment translates into safety culture outcomes. Consequently, while the overall pooled estimate indicates a positive association, it should be interpreted as a general trend rather than a precise effect size applicable to all settings.

Strengths of the Study

This study possesses several notable strengths. First, it represents one of the few meta-analytic syntheses specifically examining the relationship between nursing empowerment and patient safety culture, providing quantitative evidence of the strength and consistency of this association across diverse healthcare contexts. Second, the inclusion of subgroup analyses examining psychological empowerment, structural empowerment, and empowerment programs separately provides nuanced insights into which empowerment dimensions most strongly predict patient safety culture outcomes. Third, since the pooled effect size remained statistically significant throughout all rounds of the leave-one-out method, the sensitivity analysis validated the overall findings' robustness. Fourth, the evaluation of publication bias using Egger's test and funnel plot visualisation offers assurance that the reported effects are not significantly impacted by selective reporting. Lastly, the thorough search approach across three significant databases improves the dependability and generalisability of the findings.

Limitations

Several limitations should be considered when interpreting the findings of this review. First, the number of included studies was relatively small (k =7), and the sample sizes varied considerably across studies (range: 61 to 225 participants). This limited the statistical power for certain subgroup analyses. Second, there was substantial heterogeneity among the included studies in terms of intervention design, outcome measures, and empowerment conceptualisations. Although subgroup analysis partially explained this variability, the extremely high heterogeneity (I² ≈ 98%) indicates that substantial unexplained differences remain across studies. This limits the precision and generalisability of the pooled effect estimate and suggests that findings should be interpreted with caution. The sensitivity analysis indicated that excluding Umar et al. [6] led to a notable reduction in heterogeneity (I² decreased to 94.41%), suggesting that this study contributed disproportionately to between-study heterogeneity. Third, the majority of included studies employed cross-sectional designs, which limits the ability to establish causal relationships between empowerment and patient safety culture. Fourth, all studies relied on self-report measures for both empowerment and patient safety culture outcomes, which may introduce common method bias and social desirability effects. Finally, the geographic distribution of included studies, while spanning six countries, was concentrated in Asia and the Middle East, with limited representation from Western European, African, or Latin American healthcare systems. Despite these limitations, the core finding, a meaningful positive link between nurse empowerment and patient safety culture, remains valid. The consistency of effects across multiple contexts (even with varied methodologies) supports the conclusion that empowerment is a robust correlate of safety culture.

## Conclusions

This systematic review and meta-analysis provide robust evidence that nursing empowerment is significantly and positively associated with patient safety culture, with a strong pooled effect size indicating that empowered nurses are more likely to perceive and contribute to a positive safety culture within their healthcare organisations. The findings suggest that psychological empowerment, characterised by nurses' intrinsic motivation, sense of competence, self-determination, and perceived impact, exerts a particularly strong influence on safety culture outcomes. Healthcare organisations seeking to enhance patient safety should prioritise strategies that foster nurse empowerment, including providing access to resources, information, and support; promoting autonomous decision-making; and creating opportunities for professional growth. The non-significant effect of empowerment programs highlights the need for more rigorously designed intervention studies that incorporate appropriate control conditions, longer follow-up periods, and objective outcome measures. Future research should employ longitudinal and experimental designs to establish causal relationships, explore potential moderating factors such as organisational culture and leadership style, and examine the mechanisms through which empowerment translates into improved safety outcomes. By investing in nurse empowerment initiatives, healthcare organisations can cultivate a workforce that is not only more satisfied and engaged but also better positioned to identify, report, and prevent patient safety incidents, ultimately contributing to improved patient outcomes and organisational performance.

## Appendices

Appendix A

Search Query

Google Scholar: “("nurse empowerment" OR "structural empowerment" OR "psychological empowerment") AND ("patient safety culture" OR "patient safety" OR "safety culture" OR "patient safety activities") AND (nurses OR "staff nurses" OR nursing) AND (hospital OR healthcare) 8830”

Pubmed: “(("Empowerment, Psychological"[Mesh] OR "nurse empowerment" OR "structural empowerment" OR "psychological empowerment")” AND “("Patient Safety"[Mesh] OR "Safety Management"[Mesh] OR "patient safety culture" OR "safety culture")” AND “("Nurses"[Mesh] OR nurses OR "staff nurses" OR nursing)” AND “("Hospitals"[Mesh] OR hospital OR healthcare)) 18”

Science Direct: “("nurse empowerment" OR "structural empowerment" OR "psychological empowerment") AND ("patient safety" OR "patient safety culture" OR "safety culture") 346”

## References

[1] Ntwiga PN, Muchara M, Kiriri P (2021). The influence of employee empowerment on competitive advantage in hospitals within Nairobi, Kenya. East Afr Health Res J.

[2] Spreitzer GM (1995). An empirical test of a comprehensive model of intrapersonal empowerment in the workplace. Am J Community Psychol.

[3] Permarupan PY, Al-Mamun A, Samy NK, Saufi RA, Hayat N (2020). Predicting nurses burnout through quality of work life and psychological empowerment: a study towards sustainable healthcare services in Malaysia. Sustain Sci.

[4] Lv M, Yang S, Lv XY, Zhang L, Chen ZQ, Zhang SX (2021). Organisational innovation climate and innovation behaviour among nurses in China: a mediation model of psychological empowerment. J Nurs Manag.

[5] Albasal NA, Eshah N, Minyawi HE, Albashtawy M, Alkhawaldeh A (2022). Structural and psychological empowerment and organizational commitment among staff nurses in Jordan. Nurs Forum.

[6] Umar E, Hamdiah D, Sulastri T (2022). Relationship between empowerment and patient safety culture among nurses in Indonesia: a cross culture study. Malays J Med Health Sci.

[7] Al-Bsheish M, Mustafa M, Ismail M, Jarrar M, Meri A, Dauwed M (2019). Perceived management commitment and psychological empowerment: a study of intensive care unit nurses’ safety. Saf Sci.

[8] Page MJ, McKenzie JE, Bossuyt PM (2021). The PRISMA 2020 statement: an updated guideline for reporting systematic reviews. BMJ.

[9] Sterne JA, Savović J, Page MJ (2019). RoB 2: a revised tool for assessing risk of bias in randomised trials. BMJ.

[10] Kim BB, Yu S (2021). Effects of just culture and empowerment on patient safety activities of hospital nurses. Healthcare (Basel).

[11] Rusdi R, Said FM, Umar NS (2024). The effect of empowerment to improve patient safety culture among hospital nurses. Int J Public Health Sci.

[12] Youssef FHM, Abdelghany AM, Mahmoud HG (2025). The relationship between structural empowerment and patient safety culture among staff nurses in Fowa Central Hospital at Kafr el-Sheikh. Mansoura Nurs J.

[13] Armellino D, Quinn Griffin MT, Fitzpatrick JJ (2010). Structural empowerment and patient safety culture among registered nurses working in adult critical care units. J Nurs Manag.

[14] Çinar F, Kutlu T (2021). The effect of structural and psychologıcal empowerment of surgıcal nurses on patıent and employee safety culture. Gevher Nesibe J Med Health Sci.

[15] Amiri M, Khademian Z, Nikandish R (2018). The effect of nurse empowerment educational program on patient safety culture: a randomized controlled trial. BMC Med Educ.

[16] Xu J, Dong Z, Xie W, Yang L, Zhou Y, Li J (2025). Nurses' burnout and patient safety culture: the moderating effect of structural empowerment. J Adv Nurs.

[17] Taketomi K, Ogata Y, Sasaki M, Yonekura Y, Tanaka M (2024). A cross-sectional study examining the relationship between nursing practice environment and nurses' psychological empowerment. Sci Rep.

[18] Al-Shomrani AZ, Hamouda GM, Abdullah N (2024). The relationship between psychological empowerment and clinical decision-making among staff nurses in governmental hospital in Al-Baha, Saudi Arabia. Cureus.

[19] Allowh SN, Malak MZ, Alnawafleh AH, Ta'Amnha M (2023). The relationship between perceived management commitment to safety, psychological empowerment, and safety performance among emergency nurses in Jordan. Int Emerg Nurs.

[20] Mihdawi M, Al-Amer R, Darwish R, Randall S, Afaneh T (2020). The influence of nursing work environment on patient safety. Workplace Health Saf.

[21] Malinowska-Lipień I, Micek A, Gabryś T (2021). Impact of the work environment on patients’ safety as perceived by nurses in Poland-a cross-sectional study. Int J Environ Res Public Health.

[22] Yu H, Keele LJ, Brom H, Pittman H, Varghese R, McHugh MD, Aiken LH (2026). The impact of Magnet recognition on nurse managers’ assessments of work environment, quality, and safety: a cross-sectional study. J Nurs Manag.

[23] Huang L, Liu M, Wang X, Hsu M (2024). Interventions to support the psychological empowerment of nurses: a scoping review. Front Public Health.

[24] Suryani L, Letchmi L, Moch B (2024). The impact of nurse empowerment program on patient safety culture in general public hospital in Indonesia. J Patient Saf Risk Manag.

[25] Yilmaz A, Duygulu S (2021). Developing psychological empowerment and patient safety culture: a pre-experimental study. J Basic Clin Health Sci.

[26] Zhou B, Liu L (2026). A meta-analysis of factors influencing nurses' perceptions of patient care safety culture. Medicine (Baltimore).

